# Comparisons of the Nonlinear Relationship of Cerebral Blood Flow Response and Cerebral Vasomotor Reactivity to Carbon Dioxide under Hyperventilation between Postural Orthostatic Tachycardia Syndrome Patients and Healthy Subjects

**DOI:** 10.3390/jcm9124088

**Published:** 2020-12-18

**Authors:** Shyan-Lung Lin, Shoou-Jeng Yeh, Ching-Kun Chen, Yu-Liang Hsu, Chih-En Kuo, Wei-Yu Chen, Cheng-Pu Hsieh

**Affiliations:** 1 Department of Automatic Control Engineering, Feng Chia University, Taichung 40724, Taiwan; chingkchen@fcu.edu.tw (C.-K.C.); hsuyl@fcu.edu.tw (Y.-L.H.); cekuo@fcu.edu.tw (C.-E.K.); crazystudio9951@gmail.com (W.-Y.C.); sy850903@gmail.com (C.-P.H.); 2 Department of Neurology, Cheng Ching General Hospital, Taichung 41283, Taiwan; seanyeh1011@hotmail.com

**Keywords:** POTS, cerebral blood flow, carbon dioxide, hyperventilation, cerebral vasomotor reactivity

## Abstract

Postural orthostatic tachycardia syndrome (POTS) typically occurs in youths, and early accurate POTS diagnosis is challenging. A recent hypothesis suggests that upright cognitive impairment in POTS occurs because reduced cerebral blood flow velocity (CBFV) and cerebrovascular response to carbon dioxide (CO_2_) are nonlinear during transient changes in end-tidal CO_2_ (P_ETCO_2__). This novel study aimed to reveal the interaction between cerebral autoregulation and ventilatory control in POTS patients by using tilt table and hyperventilation to alter the CO_2_ tension between 10 and 30 mmHg. The cerebral blood flow velocity (CBFV), partial pressure of end-tidal carbon dioxide (P_ETCO_2__), and other cardiopulmonary signals were recorded for POTS patients and two healthy groups including those aged >45 years (Healthy-Elder) and aged <45 years (Healthy-Youth) throughout the experiment. Two nonlinear regression functions, Models I and II, were applied to evaluate their CBFV-P_ETCO_2__ relationship and cerebral vasomotor reactivity (CVMR). Among the estimated parameters, the curve-fitting Model I for CBFV and CVMR responses to CO_2_ for POTS patients demonstrated an observable dissimilarity in CBFV_max_ (*p* = 0.011), mid-P_ETCO_2__ (*p* = 0.013), and P_ETCO_2__ range (*p* = 0.023) compared with those of Healthy-Youth and in CBFV_max_ (*p* = 0.015) and CVMR_max_ compared with those of Healthy-Elder. With curve-fitting Model II for POTS patients, the fit parameters of curvilinear (*p* = 0.036) and P_ETCO_2__ level (*p* = 0.033) displayed significant difference in comparison with Healthy-Youth parameters; range of change (*p* = 0.042), P_ETCO_2__ level, and CBFV_max_ also displayed a significant difference in comparison with Healthy-Elder parameters. The results of this study contribute toward developing an early accurate diagnosis of impaired CBFV responses to CO_2_ for POTS patients.

## 1. Introduction

Postural orthostatic tachycardia syndrome (POTS) is typically found in youths and is frequently accompanied by numerous symptoms and comorbidities; therefore, early accurate diagnosis of POTS is challenging. Nowadays, POTS is recognized as an autonomic dysfunction that affects the flow of blood through the body and consequently causes dizziness in the presence of orthostatic intolerance; however, the palpitations of postural tachycardia usually dominate, and in most patients, POTS is commonly diagnosed by cardiologists [1,2]. Unlike Parkinson’s disease, diabetes, or other neurological disorders that are mostly associated with aged patients [3,4,5], POTS usually affects young individuals and is characterized by sudden-onset idiopathic pandysautonomia with prevailing hyperadrenergic circulatory symptoms and abnormal orthostatic heart rate (HR) acceleration [6].

Clinical and laboratory indices that often support POTS diagnosis are orthostatic HR during the first minute of head-up tilt (HUT), spectral powers of ECG during HUT, severity of orthostatic dizziness, fatigue, palpitations, shortness of breath, and autonomic deficit [7]. According to a recent hypothesis, upright cognitive impairment in patients with POTS is caused by reduced cerebral blood flow velocity (CBFV), and studies have shown that CBFV decreases excessively during 70° tilt in a minority of patients with intermittent hyperpnea/hypocapnia [8].

The sensitivity of cerebral blood flow (CBF) to carbon dioxide (CO_2_) is a unique mechanism of the cerebral blood vessel structure, and this response can be quantified to determine the response of cerebral vasomotor reactivity (CVMR) [9,10]. CVMR to CO_2_ is quantified for evaluating cerebrovascular function in clinical applications to study hypertension, stroke, heart failure, and some other disorders [11,12,13,14,15], but limited studies are available on POTS or diseases related to autonomic dysfunction.

CBFV is affected by dynamic perturbations in blood pressure within the autoregulatory range [16]. Furthermore, recent studies have observed a nonlinear CBFV response to CO_2_. Nevertheless, those experiments have mostly been performed in patients with steady-state clinical values or healthy subjects [17,18,19,20,21], and further study is still needed to clarify the interaction of CBFV and CO_2_, especially the disparities of the cerebral regulation mechanism between healthy subjects and patients with autonomic dysfunction.

To verify the hypothesis that the relationship between CBFV and end-tidal CO_2_ (P_ETCO_2__) is nonlinear during transient changes in P_ETCO_2__ (partial pressure of end-tidal carbon dioxide), a previous study was conducted with a period of voluntary hyperventilation, followed by rebreathing, to obtain a wide range of changes in P_ETCO_2__ to assess CVMR under breath-by-breath conditions [11]. During rebreathing, the CBFV-P_ETCO_2__ response was sigmoidal below a noticeable threshold P_ETCO_2__, increasing from a hypocapnic minimum to a hypercapnic maximum. Another study used a sigmoid curve minimizing the sum of squares for nonlinear regression to model the aforementioned relationship [22].

Earlier researches investigating the characteristics of CVMR have demonstrated that the relationship between cerebral blood flow and carbon dioxide is nonlinear and that this relationship is affected by CO_2_-induced changes in arterial blood pressure [10,23,24,25,26]. Despite the successful demonstration of applying a nonlinear regression function to model the CBFV-P_ETCO_2__ relationship for healthy young subjects, however, no previous study was found to utilize the nonlinear modeling method as a tool to further investigate the interaction of CBFV response to CO_2_ for POTS patients or subjects with autonomic dysfunction.

Nowadays, more attention has been paid in POTS by researchers and doctors during the past decades due to the rapidly increased cases around the world, with an estimated one million in the U.S. alone. While most studies have focused on its clinical presentation, assessment, etiology, management, and treatment strategies [2,27,28], only limited studies can be found in investigating its dissimilarity from healthy people in CA mechanism or other cerebral blood flow responses. An experimental study [29], which was considered to be the first time in humans, measured dynamic cerebral autoregulation using Doppler ultrasonography to provide new insights into cerebral blood flow (CBF) regulation during orthostatic stress and to report comparable percentage changes in the internal carotid and middle cerebral artery (MCA) velocity with head-up tilt associated with CO_2_ reductions. Under the hypothesis that POTS is mainly initiated by reduced cerebral blood flow, an earlier study [8] also used transcranial doppler ultrasound (TCD) to investigate the oscillatory CBFV in POTS patients with the relationship of measured arterial pressure, heart rate, CBFV, and P_ETCO_2__ to graded changes in tilt angels. However, no previous study was found to utilize the nonlinear modeling method as a tool to investigate the interaction of CBFV response to CO_2_ for POTS patients.

Previously, we have conducted experiments with tilt table in elderly subjects with Parkinson’s disease to study their cardiorespiratory signals and cerebral autoregulation based on CO_2_ reactivity [30,31]. The nonlinear relationship of cerebrovascular responses with CO_2_ was modeled using regression functions that have been applied earlier [11,22] in healthy subjects under a wide range of changes in P_ETCO_2__.

In the current study, we aimed to develop a method that can be clinically applied and conveniently processed to assess CVMR under breath-to-breath conditions under hyperventilation. Furthermore, we aimed to reveal the interaction between cerebral autoregulation and ventilatory control of patients with POTS during transient changes in P_ETCO_2__. HR, arterial blood pressure (ABP), CBFV, P_ETCO_2__, and breathing airflow were recorded for each subject throughout the experiment. The resultant percentage changes with respect to baseline CBFV values and CVMR values were calculated and two logistic regression functions [11,22] were applied for nonlinear regression of CBFV–P_ETCO_2__ responses during the hyperventilation phase. The current paper provides a novel study to utilize nonlinear sigmoidal models for investigating the CBFV and CVMR responses to CO_2_ for patients with POTS and further comparison with those for the control groups of healthy subjects.

## 2. Materials and Methods

**Figure 1:**
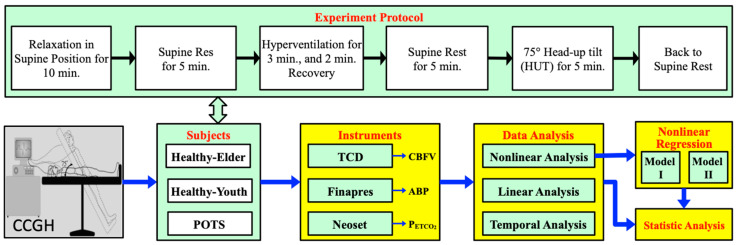
Schematic representation of the experiment protocol, subjects, instruments, and data analysis methods.

In Figure 1, the framework of the current study is schematized as a block diagram. The experiments were conducted at Neurology Diagnosis and Evaluation Center of Cheng-Ching Hospital (CCH, Taichung, Taiwan). The study complies with the human subject protection regulations of the Taiwan Ministry of Health and Welfare and received approval from the institutional review board (CCGH IRB HP200006).

### 2.1. Subjects

The study subjects were classified into the following groups: patients with POTS, healthy individuals aged <45 years (Healthy-Youth), and healthy individuals aged >45 years (Healthy-Elder). All healthy subjects had no history of cardiovascular, respiratory, or neurological conditions. The basic information of the subjects is shown in Table 1.

The patients with POTS were first diagnosed based on their clinical history and clinical examination with the symptoms of dizziness or lightheadedness, fainting, heart palpitations, headaches, shaking and sweating, shortness of breath, chest pain, poor sleep, weakness, and fatigue. Further testing was performed with the tilt table test at the Neurology Diagnosis and Evaluation Center of CCH. The POTS was diagnosed if the patient’s heart rate increased by 30 beats per minute (bpm) or measured with over 120 bpm within 5–10 min of head-up tilt (HUT). The test was also accompanied with the measurement of heart rate and blood pressure to confirm the diagnosis. However, varieties of autonomic tests were also employed to exclude autonomic disorders including orthostatic hypotension, hyperthyroidism, and rare endocrine conditions that might underlie the symptoms. All the healthy subjects and POTS patients recruited for the study had no history of cardiovascular, respiratory, hypertensive, diabetic, or other neurological conditions. The basic information of the subjects is shown in Table 1.

### 2.2. Apparatus

Continuous ABP and HR were recorded using Finapres (Model 2300, Ohmeda, Englewood, CO, USA) on the right-hand middle finger of each subject (Figure 1). The finger was held at the level of the subject’s heart including during HUT. The Finapres device used in this study was fully automated to adjust the pressure according to volume changes in the finger artery. However, because of the adjustment in movement, servo components were introduced into the recorded data. These servo components were removed using special techniques outlined in a previous study [32]. CBFV was measured using a transcranial Doppler ultrasound (TCD, EME TC2020, Nicolet Instruments, Warwick, UK) isolated at 5 MHz over the temporal window by using an elastic headband. Continuous P_ETCO_2__ and airflow signals were recorded using capnography (Neoset, FS-01382, SPEGAS Industries Ltd., Jerusalem, Israel).

HR, ABP, CBFV, P_ETCO_2__, and airflow of each subject were measured throughout the experiment, sampled at 60 Hz, and recorded simultaneously to a signal processing platform with LabVIEW^®^ for later offline analysis.

### 2.3. Experiment Protocol

All subjects were examined on a tilt-table to determine whether they could change their position from supine to 75° head-up within 4 s. Subjects first relaxed in the supine position for 10 min, and CBFV, ABP, HR, P_ETCO_2__, and airflow were recorded continuously throughout the protocol, which was as follows:

1. First, baseline data of subjects at supine rest were recorded for 5 min after 10 min of relaxation.
2. Then, the subject underwent voluntary hyperventilation in the supine position, with the breathing pattern of inhalation and exhalation for 1 s each.
3. After 3 min of hyperventilation, the subject was allowed to breathe normally for 2 min.
4. After 5 min of supine rest, the subject was tilted head-up by 75° for 10 min while breathing normally.
5. At the end of the HUT, the subject was then returned to the supine resting position.

### 2.4. Nonlinear Regressing Models for Cerebrovascular Responses

The mean values of ABP, systolic arterial pressure (SAP), HR, CBFV, breathing rate (BR), and P_ETCO_2__ were calculated and categorized for each group under the specified three phases, namely supine, hyperventilation, and HUT.

CBFV is significantly affected by ABP; however, clinical TCD assessment of CVMR generally uses linear regression of CBFV vs. P_ETCO_2__ under steady-state condition. To reveal the interaction between cerebral autoregulation and ventilatory control in patients with POTS, the resultant percentage changes with respect to baseline CBFV values and CVMR were calculated, and logistic regression used to reveal the cerebrovascular responses to CO_2_ during their hyperventilation phase.

To conduct the data analysis for cerebrovascular responses in the current study, CBFV was calculated based on percent changes using Equation (1).

(1) CBFV%=[(x−y)/y]×100%,

where *x* is the CBFV value, and *y* is the corresponding baseline, which is the average value at rest. CBFV is expressed as percentage change from the mean minimum value observed during the hyperventilation phase.

#### 2.4.1. Logistic Function Model I

In an earlier research, a nonlinear logistic curve fitting function for parameter identification was used by Battisti-Charbonney et al. [22] to quantify the CBFV-P_ETCO_2__ relationship for young healthy subjects. Below an identified P_ETCO_2__ threshold that was derived from straight-line fitting of the MAP- P_ETCO_2__ relationship, the CBFV response to P_ETCO_2__ was fitted using a sigmoid model:

(2) $$\mathrm{Model}\;I:\;f=\mathrm{CBFV}=a+\frac{b}{1+e^{-\left(x-c\right)/d}}$$

where *f* represents a percentage change in CBFV; *x* represents P_ETCO_2__ with units of mmHg; *a* is the minimum CBFV% of the hypocapnic region; *b* is the maximum CBFV% value; *c* is the midpoint value of CBFV%; and *d* is the range of the linear portion of the curve. The CVMR can further be derived by taking the derivative of Equation (2), that is, CVMR = *f*′. The first-order derivative of the logistic function was calculated using the following equation:

(3) $$f^{\prime }=\mathrm{CVMR}=\frac{\frac{b}{d}\cdot e^{-\left(x-c\right)/d}}{{\left\{1+e^{-\left(x-c\right)/d}\right\}}^{2}}$$

**Figure 2:**
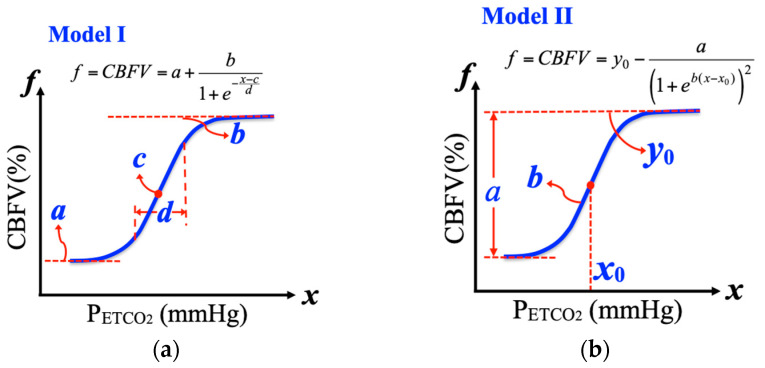
Schematic representation of the logistic function Models I and II, each with four parameters to be identified. (**a**) Model I: *f* represents a percentage change in CBFV, *x* represents P_ETCO_2__ with units of mmHg, *a* is the minimum CBFV% of the hypocapnic region, *b* is the maximum CBFV% value, *c* is the midpoint value of CBFV%, and *d* is the range of the linear portion of the curve. (**b**) Model: *f* represents percentage change in CBFV and *x* represents P_ETCO_2__, *a* represents the range of change in CBFV%, *y*_0_ is the maximum value of CBFV during hypocapnia, *x*_0_ is the level of P_ETCO_2__ where the first-order derivative of the logistic function is maximal, and *b* is related to the overall curvilinear properties of the sigmoidal curve.

This derivative function yields the specific CO_2_ sensitivity (or CVMR) for each P_ETCO_2__. A schematic representation of a logistic function with four parameters (*a*, *b*, *c*, and *d*) to be identified is shown in Figure 2 (left). In Figure 2a, the typical data of logistic regression of percent changes in CBFV to changes in P_ETCO_2__ in one specific subject are also fitted with four parameters and CVMR.

#### 2.4.2. Logistic Function Model II

In an experimental research with healthy subjects, a modified rebreathing protocol [11] was used to verify the nonlinear relationship of CBFV- P_ETCO_2__ under a wide range of changes in P_ETCO_2__, in which Claassen et al. [11] used another four-parameter logistic function for curve fitting:

(4) $$\mathrm{Model}\;\mathrm{II}:\;f=\mathrm{CBFV}=y_{0}-\frac{a\cdot }{1+e^{\left[b\cdot \left(x-x_{0}\right)\right]}},$$

where *f* represents percentage change in CBFV and *x* represents P_ETCO_2__. A schematic representation of a logistic function with four parameters (*a*, *b*, *y*_0_, and *x*_0_) to be identified is shown in Figure 2b. As in the current study, in Equation (3) of Model II, parameter *a* represents the range of change in CBFV%, *y*_0_ is the maximum value of CBFV during hypocapnia, *x*_0_ is the level of P_ETCO_2__ where the first-order derivative of the logistic function (the slope of the curve) is maximal, and *b* is related to the overall curvilinear properties of the sigmoidal curve. The use of this model is mainly based on the fact that model parameters of the selected logistic function have clear physiological implications [33] and curvilinear distribution for breath-by-breath changes in CBFV vs. P_ETCO_2__.

The first-order derivative of the logistic function can also be obtained as follows:

(5) $$f^{\prime }=\mathrm{CVMR}=f^{\prime }\left(x\right)=\frac{a\cdot b\cdot e^{\left[b\cdot \left(x-x_{0}\right)\right]}}{{\left\{1+e^{\left[b\cdot \left(x-x_{0}\right)\right]}\right\}}^{2}}.$$

At *x* = *x*_0_, the CVMR of Equation (5) becomes maximal (CVMR_max_), and Equation (5) becomes (*a* × *b*)/4.

The curve fitting of the CBFV-P_ETCO_2__ relationship applied by either Model I or Model II was performed by minimizing the sum of squares for nonlinear regression with the Marquardt–Levenberg algorithm for model parameter identification (IBM SPSS).

### 2.5. Statistical Analysis

With the characteristics of unequal sample size for three subject groups in this study (POTS: n = 60; Healthy-Youth: n = 13; Healthy-Elder n = 10) and the Mann–Whitney U nonparametric tests, a method without the need for normal distribution assumption was adopted for the statistical analysis of significance in nonlinear fit parameters between any two independent groups (POTS vs. Healthy-Youth, or POTS vs. Healthy-Elder) from three subject groups. The analysis of significance for mean values between baseline (rest) and experiment phases (hyperventilation and tilt-up) within each subject group was evaluated using one-way repeated-measures ANOVA. Both of these tests were performed using SPSS. Data of the subject groups are presented as mean and standard deviation (SD), and p < 0.05 was considered statistically significant. The estimation of logistic function parameters of the curve fit results are presented as mean, SD, and CV.

## 3. Results

The mean values of cardiorespiratory signals including P_ETCO_2__, MHR (mean heart rate), MSBP (mean systolic blood pressure), MABP (mean arterial blood pressure), MBR (mean breathing rate), and MCBFV (mean cerebral blood flow velocity) during data acquisition and the experiment protocol are shown in Table 2 for the three subject groups included in the study. The significant results for patients with POTS from Table 2 are excerpted as follows:

1. Compared with the Healthy-Youth group, the POTS group was significantly different in terms of:
  - During supine (rest)—P_ETCO_2__, MHR, MSBP, MABP, and MBR.
  - During hyperventilation —MSBP and MABP.
  - During tilt-up—P_ETCO_2__, MHR, MSBP, and MABP.
2. Compared with the Healthy-Elder group, the POTS group was significantly different in terms of:
  - During supine (rest)—MHR, MSBP, MABP, and MCBFV.
  - During hyperventilation—MHR, MSBP, MABP, MBR, and MCBFV.
  - During tilt-up—MSBP.
3. Compared with its own baseline data, the POTS group was significantly different in terms of:
  - During hyperventilation —P_ETCO_2__, MBR, and MCBFV.
  - During tilt-up—P_ETCO_2__, MHR, and MCBFV.

During the supine rest, the POTS showed significant lower P_ETCO_2__ (26.86 ± 3.50 mmHg) and higher MHR (74.96 ± 11.38 beat/min) and MBR (15.39 ± 3.85 breath/min) than the Healthy-Youth of similar age, and this clinically verified that the patients with POTS were usually diagnosed with the symptoms of recurring hyperventilation, heart palpitations, and shortness of breath. The blood pressure of the POTS group including MSBP (104.37 ± 14.12 mmHg) and MABP (77.96 ± 9.76 mmHg), also exhibited lower than two healthy subject groups during the rest condition. Despite having lower P_ETCO_2__, the POTS group posed a higher MCBFV than both control groups of healthy subjects although no statistical significance was displayed.

During the hyperventilation phase and in comparison, to the Healthy-Youth, no significant difference in mean values of signals for the POTS was found except in MSBP and MABP.

During their tilt up position, as we can see from Table 2, the patients with POTS again exhibited significant higher heart rate (MHR = 85.13 ± 18.27 beat/min) than the two healthy subject groups, and this is also in consistent with their clinical diagnosis, where a change from lying to standing causes an abnormally large increase in heart beat rate. The POTS also showed a lower P_ETCO_2__ (24.67 ± 4.11 mmHg), MSBP, and MABP in comparison to the Healthy-Youth group at the tilt up position.

### 3.1. Mean Temporal Responses under Hyperventilation

**Figure 3:**
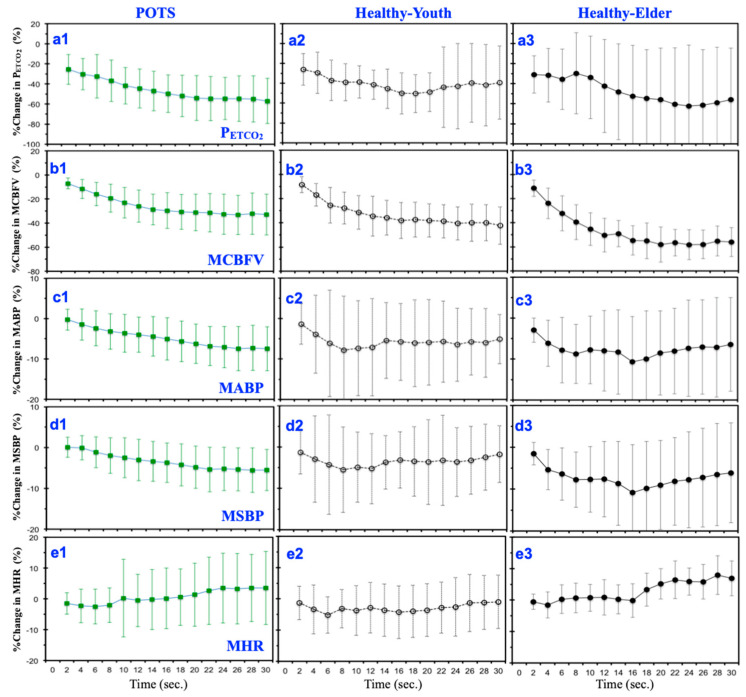
Mean percentage changes in P_ETCO_2__ (**a1**–**a3**), MCBFV (**b1**–**b3**), MABP (**c1**–**c3**), MSBP (**d1**–**d3**), and MHR (**e1**–**e3**) under an initial 30 s of hyperventilation (15 breathing cycles) for the three groups: POTS (left column, **a1**–**e1**), Healthy-Youth (middle column, **a2**–**e2**), and Healthy-Elder (right column, **a3**–**e3**).

To demonstrate the breath-to-breath transient responses of cardiopulmonary signals under hyperventilation for each breathing cycle, the corresponding value for P_ETCO_2__ and the mean values for CBFV, ABP, SBP, and BR were measured. Figure 3 shows the percentage changes in P_ETCO_2__, MCBFV, MABP, MSBP, and MBR from the baseline under the first 30 s of hyperventilation, in which the steepest changes in CBFV and P_ETCO_2__ were observed for the three experimental groups.

During hyperventilation, the temporal responses of changes in P_ETCO_2__ (Figure 3(a1)) and MCBFV (Figure 3b) did not show significant dissimilarity for the POTS group, and all appeared to decrease continuously. In comparison with the POTS (Figure 3(a1) and Healthy-Elder (Figure 3(a3)) groups, the change in P_ETCO_2__ (Figure 3(a2) for the Healthy-Youth group) showed steady variation after few breaths.

On the basis of changes in MABP (Figure 3(c1)) and MSBP (Figure 3(d1)) for patients with POTS, not much can be inferred, although they both showed a constantly increasing rate of change (drop) in blood pressure compared with healthy subjects (Healthy-Youth: Figure 3(c2,d2); Healthy-Elder: Figure 3(c3,d4).

The percentage change patterns in MHR did not show a substantial difference among the POTS (Figure 3(e1)) and Healthy-Youth (Figure 3(e2)) groups. However, a more rapid increase in MHR was observed in the POTS group than in the Healthy-Youth (Figure 3(e2)).

**Figure 4:**
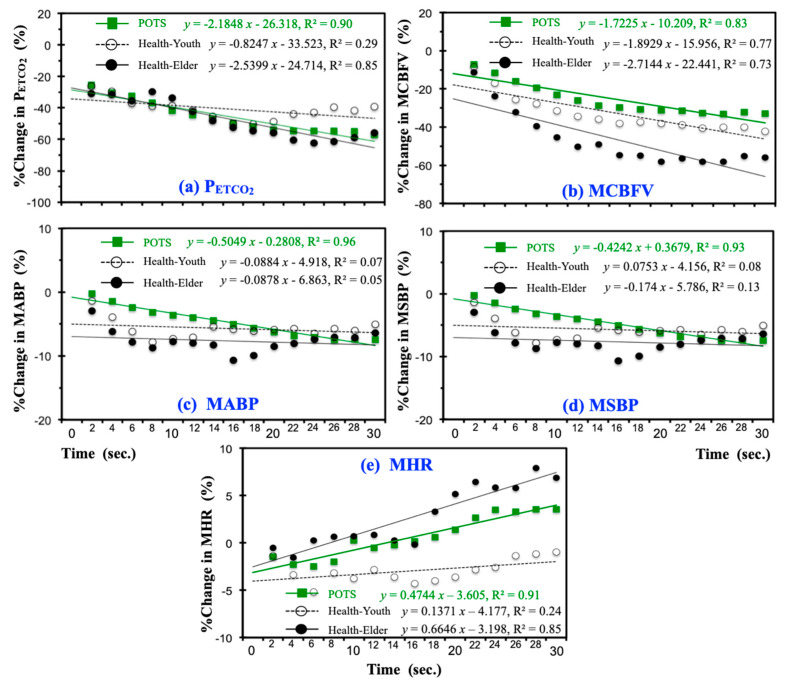
Mean percentage changes in (**a**) P_ETCO_2__, (**b**) MCBFV, (**c**) MABP, (**d**) MSBP, and (**e**) MHR during the initial 30 s (15 breathing cycles) of hyperventilation for the three groups with linear regression.

During the hyperventilation phase of the experiment, the participants’ CO_2_ level descended abruptly. Although the CBFV response to CO_2_ is nonlinear, to determine the alterations of sensitivity in CBFV, CO_2_, and blood pressure for patients with POTS in comparison with healthy subjects, we further studied their mean percentage changes in P_ETCO_2__ (Figure 4a), MCBFV (Figure 4b), MABP (Figure 4c), MSBP (Figure 4d), and MHR (Figure 4e) in the initial 30 s (15 breathing cycles) of hyperventilation in the three groups through linear regression.

In Figure 4a, while the change in P_ETCO_2__ for patients with POTS seemed comparable to healthy elders, it decreased more abruptly than in healthy youths (POTS: slope = −2.1848% mmHg·s^−1^; Healthy-Youth: slope = −0.8247% mmHg·s^−1^; Healthy-Elder: slope = −0.8247% mmHg·s^−1^) during hyperventilation. However, in Figure 4b, the changes in MCBF of the POTS and Healthy-Youth groups showed similar descending rates (POTS: slope = −1.7225% mmHg·s^−1^; Healthy-Youth: slope = −1.8929% mmHg·s^−1^), but were separated by a nearly constant gap of approximately 10%.

CBFV is affected by dynamic perturbations in blood pressure within the autoregulatory range. Furthermore, as shown in Figure 4c, the POTS group displayed the steepest decrease in changes in MABP (POTS: slope = −0.5049% mmHg·s^−1^) compared with the two healthy groups (Healthy-Youth: slope = −0.0884% mmHg·s^−1^; Healthy-Elder: slope = −0.0878% mmHg·s^−1^), which both showed approximately constant change rate, but that of the Healthy-Youth group was above that of the Healthy-Elder group, with a nearly 5% difference. Changes in MSBP in Figure 4c appeared to have similar patterns as MABP in Figure 4d.

In Figure 4e, regression of the change in MHR for patients with POTS lay between that for two healthy groups. The POTS group was observed with a more speedily increased heart rate (POTS: slope = 0.4744% bpm·s^−1^) than the Healthy-Youth group (Healthy-Youth: slope = −0.5049% bpm·s^−1^) under hyperventilation.

### 3.2. Linear Responses of Cardio-Respiratory Signals to Carbon Dioxide under Hyperventilation

**Figure 5:**
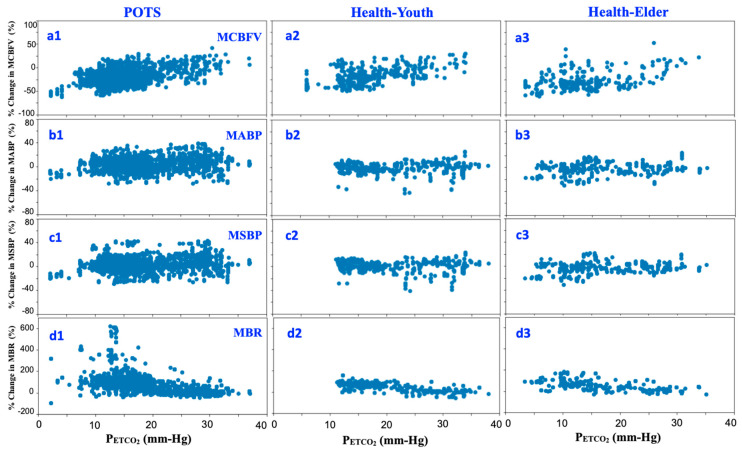
Linear analysis of percentage change in cardiorespiratory signals based on the variation in P_ETCO_2__: (**a1**–**a3**) MCBFV; (**b1**–**b3**) MABP; (**c1**–**c3**) MSBP; and (**d1**–**d3**) MBR during the initial 30 s of hyperventilation for the three subject groups, (1) POTS; (2) Healthy-Youth; (3) Healthy-Elder.

To illustrate the correlation of the measured CBFV, blood pressure, and respiratory signals to the variation in CO_2_ induced through hyperventilation, Figure 5 records all the breath-to-breath mean values of the percentage change in MCBFV (Figure 5(a1–a3)), MABP (Figure 5(b1–b3)), MSBP (Figure 5(c1–c3)), and MBR (Figure 5(d1–d3)) from the baseline based on the variation of P_ETCO_2__ for the three experimental groups.

**Figure 6:**
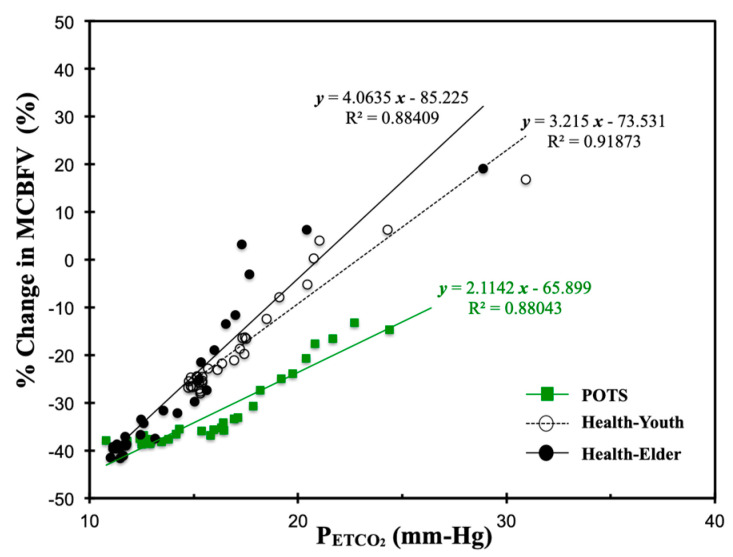
Linear analysis of averaged percentage change in MCBFV to CO_2_ for the three subject groups during 30 s of hyperventilation.

The breath-to-breath mean values of the change in MCBFV from the baseline based on the variation in P_ETCO_2__ for the three experimental groups (Figure 5(a1–a3)) did not depict the intrinsic information regarding the relationship between MCBFV and P_ETCO_2__. To further examine the sensitivity for the change in MCBFV to the variation in P_ETCO_2__, the data of each group were averaged and analyzed using linear regression (Figure 6).

### 3.3. Nonlinear Regression of Cerebrovascular Response to Carbon Dioxide under Hyperventilation

This section presents the curve fit results for the three groups obtained by applying the nonlinear logistic regression function of Models I and II, which were earlier used by Battisti-Charbonney et al. [22] and Claassen et al. [11], respectively, to quantify the CBFV–P_ETCO_2__ relationship for young healthy subjects. For each model, four logistic function (*f*) parameters (Model I: *a*, *b*, *c*, and *d*; Model II: *a*, *y*_0_, *x*_0_, and *b*) were estimated and the CVMR_max_ values were tabulated (Table 3 and Table 4 for Models I and II, respectively) as averaged group results after the exclusion of data outliers. Furthermore, the nonlinear fitted curves with two models (Figure 5(a1–a3,b1–b3) for Models I and II, respectively) for percentage changes in CBFV within the P_ETCO_2__ range during hyperventilation are shown along with their CVMR results (*f’*, slope of its corresponding curve) for the three subject groups.

#### 3.3.1. Curve-Fitting and Model Parameters of Logistic Function Model I

**Figure 7:**
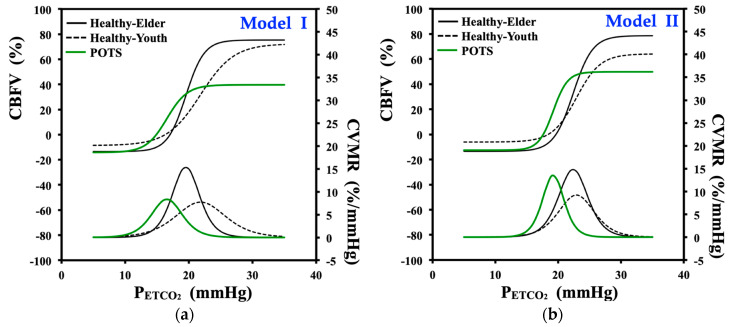
Nonlinear curve fit results of percentage change in CBFV responses to CO_2_ (P_ETCO_2__) during hyperventilation for the three subject groups: (**a**) nonlinear regression with curve-fitting Model I of Equation (2); (**b**) nonlinear regression with Model II of Equation (3).

The curve fit results of the CBFV response to CO_2_ with logistic function Model I of Equation (2) for the three subject groups are shown in Table 3. The averaged fitting parameters of the CBFV–P_ETCO_2__ relationship for the POTS group were −14.02 ± 14.11% for CBFV_min_ (*a*), 52.53 ± 31.09% for CBFV_max_ (*b*), 16.38 ± 3.48 mmHg for mid- P_ETCO_2__ (*c*), 1.47 ± 1.18 mmHg for the entire P_ETCO_2__ range (*d*), and 12.65 ± 8.10%/mmHg for CVMR_max_. Figure 7a shows the group averaged results of fitting CBFV and CVMR responses to P_ETCO_2__ during hyperventilation for the three groups. All CBFV values were converted to percentage change with respect to the baseline data.

The steep ranges of the sigmoidal curve for the POTS, Healthy-Youth, and Healthy-Elder groups were 10–20, 18–30, and 15–22 mmHg P_ETCO_2__, respectively (Figure 7a). The most noteworthy observation from Figure 5(a1–a3) is that the CBFV_max_ (*b*) of the sigmoidal curve for the POTS group (CBFV_max_ = 52.53 ± 31.09%) was far below the levels for healthy subjects (Healthy-Youth: CBFV_max_ = 80.95 ± 28.80%; Healthy-Elder: CBFV_max_ = 80.95 ± 28.80%). Moreover, this suggests that for POTS patients, a rather low change in CBFV was assessed at the beginning of the hyperventilation when the P_ETCO_2__ level started to decrease rapidly. For the POTS group, the decreasing CBFV due to descending P_ETCO_2__ was not significant until a P_ETCO_2__ level lower than that of healthy subjects was reached. Figure 7a shows that the mid-P_ETCO_2__ (*c*) of the sigmoid curve for the POTS group (mid- P_ETCO_2__ = 16.38 ± 3.48 mmHg) was shifted to the left (small value) compared with healthy subjects (Healthy-Youth: mid-P_ETCO_2__ = 21.83 ± 5.84 mmHg; and Healthy-Elder: mid-P_ETCO_2__ = 19.52 ± 7.58 mmHg).

As expected, Figure 7a shows that the sigmoid curve of the CBFV–P_ETCO_2__ relationship for the Healthy-Youth group was smoother than that for the Healthy-Elder group, in which change in CBFV responded sharply to variation in CO_2_ with a lower P_ETCO_2__ range (Healthy-Youth: P_ETCO_2__ range = 2.62 ± 1.92 mmHg; Healthy-Elder: P_ETCO_2__ range = 19.52 ± 7.58 mmHg).

The CVMR curves in Figure 7a also characterize that the impaired cerebral vasomotor response of POTS patients was notably diverse from that of healthy youths, with late responses to a decreased CO_2_ level during hyperventilation, although the two groups had similar CVMR_max_ levels (POTS: CVMR_max_ = 12.65 ± 8.10%·mmHg^−1^; Healthy-Youth: CVMR_max_ = 10.92 ± 6.92%·mmHg^−1^).

#### 3.3.2. Curve-Fitting and Model Parameters of Logistic Function Model II

In Table 4, the curve fit results of the CBFV response to CO_2_ with the logistic function Model II of Equation (4) for the three subject groups are presented. The averaged fitting parameters for the POTS group in the CBFV–P_ETCO_2__ relationship were 62.42 ± 31.94% for the range of change (*a*), 49.8 ± 32.93% for CBFV_max_ (*y*_0_), 19.2 ± 4.83 mmHg for the P_ETCO_2__ level (*y*_0_), 0.87 ± 0.54 mmHg^−1^ for curvilinear (*b*), and 12.49 ± 9.18%/mmHg for CVMR_max_. Figure 7b displays the group average results of fitting CBFV and CVMR responses to P_ETCO_2__ during hyperventilation for the three groups.

The steep ranges of the sigmoidal curve for POTS, Healthy-Youth, and Healthy-Elder group were 10–20, 20–30, and 18–25 mmHg P_ETCO_2__, respectively (Figure 7b). Similar to the curve fit results of Model I, Figure 5(b1–b3) again demonstrates that the CBFV_max_ (*y*_0_) of the sigmoidal curve for the POTS group (CBFV_max_ = 49.8 ± 32.93%) was located below that for healthy subjects (Healthy-Youth: CBFV_max_ = 62.13 ± 38.66%; Healthy-Elder: CBFV_max_ = 78.52 ± 28.65%). Therefore, the range of change (*a*) in CBFV for the POTS group (*a* = 62.42 ± 31.94%) was also the smallest in comparison with that of the healthy groups (Healthy-Youth: *a* = 69.89 ± 39.38%; Healthy-Elder: *a* = 92.05 ± 32.67%). Compared with healthy subjects that considered furnishing with comparative intact autonomic CBF regulation to CO_2_, POTS patients had a much lower P_ETCO_2__ at the beginning of hyperventilation. Compared with the Healthy-Elder group, the Healthy-Elder group also responded to lower P_ETCO_2__ levels with a change in CBFV, which descended sharply in the steep portion of the sigmoidal curve.

The curve-fitting parameter *b* in Model II reflects the overall curvilinear properties of the sigmoidal curve. Figure 7b shows a fit result of curvilinear as POTS (*b* = 0.87 ± 0.54) > Healthy-Elder (*b* = 0.64 ± 0.59) > Healthy-Youth (*b* = 0.47 ± 0.16).

In logistic function Model II, the CVMR of Equation (5) becomes maximal at *x* = *x*_0_. Hence, the curve-fitting parameter *x*_0_ generally reflects the location of the CVMR_max_ in Figure 7b for each subject group. The POTS group showed a similar CVMR_max_ to the older healthy group (POTS: CVMR_max_ = 12.49 ± 9.18%·mmHg^−1^; Healthy-Youth: CVMR_max_ = 7.67 ± 3.67%·mmHg^−1^; and Healthy-Elder: CVMR_max_ = 12.41 ± 8.47%·mmHg^−1^), while also demonstrating that its CVMR_max_ shifted to the lower level in P_ETCO_2__ than of the two healthy groups (POTS: *x*_0_ = 19.2 ± 4.83 mmHg; Healthy-Youth: *x*_0_ = 23.27 ± 4.58 mmHg; and Healthy-Elder: *x*_0_ = 22.37 ± 5.29 mmHg).

Similar to the curve fit results we obtained from Model I, the group average parameters with Model II also indicated that the POTS group had a lesser level of change in CBFV than both the healthy subject groups with a smaller P_ETCO_2__ range.

#### 3.3.3. Statistical Analysis of Fit Parameters for Models I and II

To analyze the fitted CBFV responses to the CO_2_ of the three experimental groups, we further applied Mann–Whitney U tests for averaged fit parameters between each of the two healthy groups and the POTS group. The statistical test results for fit parameters and CVMR_max_ are presented in Table 5 and Table 6 for the regression function Models I and II, respectively.

Using Model I (Figure 7a), we observed that P_ETCO_2__, at which CBFV reached its minimum (10–15 mmHg), was consistent with an earlier finding (10–15 mmHg) [34], but the CBFV_min_ of the POTS group in Table 5 did not show a significant difference in comparison with that of either healthy group (POTS vs. Healthy-Youth: *p* = 0.695; POTS vs. Healthy-Elder, *p* = 0.716). The plateau maximum of CBFV for the POTS group occurred at the turning point of P_ETCO_2__ ≈ 15 mmHg, in comparison with P_ETCO_2__ ≈ 30 mmHg and P_ETCO_2__ ≈ 35 mmHg for Healthy-Youth and Healthy-Elder groups, respectively (Figure 7a). Although the intrinsic mechanism remains unknown, this indicated a lower threshold CO_2_ level for the impaired CBF regulation for patients with POTS. In contrast to CBFV_min_, the CBFV_max_ of POTS (Table 5) also showed significant differences from healthy subject groups (POTS vs. Healthy-Youth: *p* = 0.011; POTS vs. Healthy-Elder, *p* = 0.003).

The CBFV responses (Figure 7a) revealed that sigmoidal curves were fitted between 10 and 20 mmHg P_ETCO_2__ for POTS, 18 and 30 mmHg P_ETCO_2__ for Healthy-Youth, and 15 and 22 mmHg P_ETCO_2__ for Healthy-Elder groups. The CBFV response of patients with POTS under hyperventilation clearly shifted leftward to a lower CO_2_ range, and this was exemplified by the fit parameter of the mid- P_ETCO_2__ (*c*) and P_ETCO_2__ range (*d*) at which the POTS displayed significant difference from the healthy youths (mid-P_ETCO_2__: *p* = 0.013, P_ETCO_2__ range: *p* = 0.023). Although the leftward shifted sigmoidal curve consequently marked the location of CVMR_max_ at a lower CO_2_ tension for the POTS group, the POTS group did not display significance in CVMR_max_ compared with the Healthy-Youth. However, the CVMR_max_ was significantly different between the POTS and Healthy-Elder groups.

Using Model II (Figure 7b), we found that P_ETCO_2__ at which the CBFV was minimum was 15–20 mmHg in comparison with 10–15 mmHg of Model I. In Table 6, we observed that the POTS group had a lower CBFV_max_, similar to Model II, than that of the two healthy groups, but only the comparison of POTS vs. Healthy-Elder revealed a statistical significance (*p* = 0.033). As the location of *x*_0_ (P_ETCO_2__ level) also indicates where the fitted sigmoidal curve is centered, the POTS group attained significant difference in *x*_0_ compared with either healthy subject group (POTS vs. Healthy-Youth: *p* = 0.033; POTS vs. Healthy-Elder, *p* = 0.033). Similar to Model I, we did not find any significance in the test of CVMR_max_ parameters (POTS vs. Healthy-Youth: *p* = 0.251; POTS vs. Healthy-Elder, *p* = 0.676), although both the location and the peak of derived CVMR curve showed its distinction from the healthy subject groups in Figure 7b.

## 4. Discussion

### 4.1. Temporal Responses under Hyperventilation

Although recent studies have revealed that the CBFV response to CO_2_ is nonlinear, linear regression was applied in Figure 4 only to indicate the changing rate in CBFV, CO_2_, and blood pressure for the three subject groups under the initial 30 s of hyperventilation, in which the steepest variation in P_ETCO_2__ and MCBFV occurred as we examined the original signals. The mean data at each breath of the POTS group (*n* = 60) still presented excellent fit results with *r*^2^ of 0.90, 0.83, 0.96, and 0.93 for mean percentage changes in P_ETCO_2__, MCBFV, MABP, and MSBP, respectively. Nevertheless, in two groups of healthy subjects that have few test participants (Healthy-Youth: *n* = 13; Healthy-Elder: *n* = 10), the linear regression cannot be performed reliably, as it yielded an *r*^2^ of 0.29, 0.77, 0.07, and 0.08 in the Healthy-Youth group and 0.85, 0.73, 0.05, and 0.13 in the Healthy-Elder group for mean percentage changes in P_ETCO_2__, MCBFV, MABP, and MSBP, respectively.

During the hyperventilation phase, the changes in CO_2_ level causes the peripheral blood vessels to dilate, and this consequently results in a decrease in BP and variance in blood flow. However, the BP responses during hyperventilation may become significant in some patients with vascular disease or diabetes, which have been clinically verified as impairment of cerebral autoregulation (CA) or autonomic neuropathy.

In the current study, all healthy subjects and POTS patients recruited for the study had no history of cardiovascular, respiratory, hypertensive, diabetic, or other neurological conditions and autonomic disorders. Hence, we considered that the BP responses to CO_2_ under hyperventilation should exhibit equal effect on the three subject groups and would not play an important factor in the study of CBFV response to CO_2_.

### 4.2. Linear Responses of Cardiorespiratory Signals to CO_2_ under Hyperventilation

A study by Claassen et al. [11] in 10 healthy youths (*n* = 10, age = 37 ± 8 years), in which the nonlinear regression function of Model II was applied, also assessed the MCBFV–P_ETCO_2__ linear relationship by estimating CVMR_0_, the linear regression slope of changes in MCBFV over the entire range of changes in P_ETCO_2__, and CVMR_1_, the linear regression slope of changes in MCBFV in the steep ranges of P_ETCO_2__ for the sigmoidal fitted curves. The slopes of the MCBFV–P_ETCO_2__ linear relationship estimated in the current study (Figure 6) were more comparable to the CVMR_0_ in an earlier study [11]. However, the range of changes in P_ETCO_2__ in the current study was between 5 and 30 mmHg caused by hyperventilation, in comparison to the wider range of CO_2_ changes (10–65 mmHg) caused by voluntary hyperventilation preceding rebreathing in the earlier study [11].

On the other hand, another experimental study conducted by Battisti-Charbonney et al. [22] in healthy human subjects (*n* = 10, age = 27 ± 5.8 years) for cerebrovascular responses to increasing CO_2_ hyperoxic and hypoxic rebreathing, where the test subjects hyperventilated to a target P_ETCO_2__ range between 20 and 25 mmHg during hyperventilation= also estimated the slope of the fitted sigmoidal curve for the middle cerebral artery flow velocity (left MCA_v_ and right MCA_v_) response to CO_2_ within hyperoxic and hypoxic ranges.

The CVMR_0_ and CVMR_1_ estimated by Claassen et al. [11] were both 5 ± 1%·mmHg^−1^. The sigmoidal slopes estimated by Battisti-Charbonney et al. [22] were 7.8 ± 0.5%·mmHg^−1^ (left MCA_v_) and 8.1 ± 1.1%·mmHg^−1^ (left MCA_v_) for the hyperoxic range and 11 ± 0.9%·mmHg^−1^ (left MCA_v_) and 10.9 ± 1.1%·mmHg^−1^ (left MCA_v_) for the hyperoxic range.

The linear relationship of MCBFV–P_ETCO_2__ (Figure 6) for the POTS group (slope = 2.1142, *r*^2^ = 0.88) appeared to be flatter than for the two healthy subject groups (Healthy-Youth: slope = 3.215, *r*^2^ = 0.92; Healthy-Elder: slope = 4.06, *r*^2^ = 0.88). Moreover, this suggested the lag of sensitivity in the regulation of the CBF response to the rapidly changed CO_2_ levels during the initial hyperventilation period. Nonetheless, the two linear regression lines of the healthy groups did not indicate a difference in the solidity of cerebral autoregulation between youths and older individuals.

In comparison with the CVMR_max_ estimated by the two nonlinear regression models, the slope of linear regression seemed to underestimate the maximal cerebral vasodilatory effect of CO_2_. Using the CVMR_max_ of the POTS group as an example, CVMR_max_ = 12.65 ± 8.10 and 12.49 ± 9.18%·mmHg^−1^ were estimated using curve-fit Models I and II, respectively, compared with slope = 2.1142%·mmHg^−1^ through linear regression.

### 4.3. Nonlinear Curve-Fitting and Model Parameters

We adopted two sigmoidal regression function models, Model I and Model II, for our CBFV response analysis during the hyperventilation phases for three subject groups, as did Claassen et al. [11] and Battisti-Charbonney et al. [22], who used healthy youths as test subjects. Although data with a sigmoidal distribution could be fitted with choices of nonlinear regression functions, the two models used in the current research and previous studies [11,22] were identified to provide clear physiological meanings of their model parameters.

During hyperventilation, the CBFV response displayed a sigmoidal function with respect to transient changes in CO_2_. The fitted curves of CBFV and CVMR responses to P_ETCO_2__ showed similar behaviors through slightly different fit parameters by two models (Figure 7). However, the statistic tests (Table 4 and Table 5) for differences between POTS group and two healthy subject groups indicated two models estimated the center of the fitted curve (Model I: mid-P_ETCO_2__ *c*; Model II: P_ETCO_2__ level *x*_0_) diverged from healthy youths with consistent significance. Under the assumption that the proposed nonlinear models accurately fitted the sigmoid CBFV response to CO_2_ and correctly described the regulation of CBFV by CO_2_ through changes in the diameter of cerebral vessels, it may be claimed that above mid-P_ETCO_2__, the center of the steep range of the sigmoidal curve, not only indicates the P_ETCO_2__ at which vessel responsiveness is at its maximum, but also the P_ETCO_2__ at which vessel diameter is at its midpoint [22].

In comparison with healthy elders, both models conveyed coherent test results on the lessened maximum CBFV (CBFV_max_) for the POTS group. The test on CVMR for the two models seemed to be a lack of significance compared with healthy youths. The CBF response to transient changes in CO_2_ showed a time delay [34]. However, identification of the accurate time delay between transient changes in CBF and P_ETCO_2__ needs complex and precise control of the CO_2_ levels and breathing process during the experiment, and the delay was only compensated manually on raw signals in the current study. This factor might have affected the estimation of CVMR with the regression models.

It is well-known that CA is very sensitive to changes in carbon dioxide. Although physiological aging is known to be associated with many changes in the cardiovascular and cerebrovascular systems and also with impairment in a number of conditions, dynamic CA was also shown to be preserved in normal subjects during orthostatic stress, but no age-related deterioration has been demonstrated in dynamic CA in normal subjects during supine rest [35]. A study that investigated the effect of aging on dynamic CA in normal subjects during orthostatic stress induced by head-up tilt also showed autoregulatory indexes were similar in younger and older subjects at all times before, during, and after tilt, although CBFV was significantly lower at rest in aged subjects [36]. However, none of the related studies were performed to demonstrate the dynamic relationship of CBFV response to CO_2_ between healthy youths and elders under hypocapnic or hypercapnic range of change in carbon dioxide, not to mention the nonlinear relationship of cerebrovascular response during transient changes in CO_2_.

An earlier study investigated the effect of aging on CA using a moving-window autoregressive moving average (MWARMA) to calculate CA as an autoregulatory index (ARMA-ARI) during hypercapnia and hypocapnia, and the results suggested that CA is not affected by healthy aging and also demonstrated that CBFV was higher and change in CBFV due to respiratory maneuver was significantly greater in the younger group compared with the aged group [37].

Meanwhile, studies investigating the effect of aerobic exercise training on CVMR also showed mixed results about aged subjects. It was observed that both hypo- and hypercapnic CVMRs were similar between endurance Masters athletes and age-matched sedentary older adults by using TCD during hyperventilation and modified rebreathing [38]. On the other hand, with similar participant groups, it was found the youth adults had lower measured CVMR than aged adults by using functional MRI during steady-state hypercapnia [39].

The steady-state level of CBF progressively decreased in normal aging adults [40]. This age-related reduction in CBF might reflect decreased cerebral metabolic rate and cerebrovascular dysfunction. Nevertheless, the objective current study was not to investigate the comparison between youths and elders, or to examine whether the age would alter the CBFV response to CO_2_. We utilized nonlinear regression functions to model the CBFV-CO_2_ relationship during transient changes in P_ETCO_2__ within hypocapnic range to further explore the nonlinear cerebral blood flow response and cerebral vasomotor reactivity to carbon dioxide between patients with POTS and healthy subjects, mainly healthy youths.

In two groups of healthy subjects that only had fewer test participants (Healthy-Youth: *n* = 13, Healthy-Elder: *n* = 10), the linear regression could not be performed reliably as it yielded an *r*^2^ of 0.29, 0.77, 0.07, and 0.08 in the Healthy-Youth group, and *r*^2^ of 0.85, 0.73, 0.05, and 0.13 in the Healthy-Elder group for mean percentage changes in P_ETCO_2__, MCBFV, MABP, and MSBP, respectively.

Indeed, current research is the first attempt to utilize nonlinear models, which were earlier applied to investigate healthy youth subjects in patients with POTS to access their dynamic CBFV response and CVMR during transient changes in P_ETCO_2__, and comparisons were performed with healthy youths and elders. Of course, we could still find the difference between healthy youths and elders based on this pioneering viewpoint. The aged group appeared to have lower mean values in P_ETCO_2__ and CBFV, and higher mean values in ABP during hyperventilation. As we can see from Figure 7, the nonlinear curve fit results of Models I and II for the Healthy-Youth posed lower CVMR with both models than Healthy-Elder, and this is consistent with the earlier finding [39] with the use of functional MRI during steady-state hypercapnia. However, both fitted sigmoidal curves also indicated that the aged group was modeled with higher CBFV_max_ and responded to lower P_ETCO_2__ level and descended more sharply in the steep portion of the sigmoidal curve. Nevertheless, the statistical significance analysis in the aging effect was beyond the scope of the current study.

## 5. Conclusions

With the designed experiment, we hypothesized that hyperventilation of room air lowered P_ETCO_2__ sufficiently to produce a maximum CO_2_-modulated vasoconstriction so that the vessel diameter could no further decrease. To reveal the hypothesized nonlinear CBFV response to CO_2_ and CVMR for POTS, experimental data of 60 patients with POTS, along with 13 healthy youths and 10 healthy elderly individuals for comparisons, were analyzed. To reveal the nonlinear relationship between CBFV responses to CO_2_, we adopted two nonlinear regression functions, Model I and Model II, for sigmoidal curve fitting during hyperventilation phases, as did Claassen et al. [11] and Battisti-Charbonney et al. [22], respectively. Although the linear regression slope of the CBFV response to CO_2_ was calculated, the linear analysis method practically underestimates the maximal cerebral vasodilatory effect of CO_2_, as shown by the estimated CVMR_max_ using either model.

Using Model I, the curve fit parameters and statistical tests indicated that significant differences in CBFV_max_, mid-P P_ETCO_2__, and P_ETCO_2__ ranged between the POTS and Healthy-Youth groups, and in CBFV_max_ and CVMR_max_ between the POTS and Healthy-Elder groups.

Using Model II, the curve fit parameters and statistical tests indicated significant differences in curvilinear and mid- P_ETCO_2__ between the POTS and Healthy-Youth groups, and in the range of change, P_ETCO_2__ level, and CBFV_max_ between the POTS and Healthy-Elder groups.

In general, compared with the healthy subjects, the fitted curves with both models for patients with POTS illustrated that the mid- P_ETCO_2__ point, the center of the steep range of the sigmoidal curve, significantly departed toward a low CO_2_ tension, and the maximum CBFV level was also attenuated. However, the estimated CVMR_max_ did not show a consistent and sufficient statistical significance between patients with POTS and healthy groups, although graphical differences were observed.

The results of the current study play an important role in the effort to develop an early accurate diagnosis for the impairment CBFV response to CO_2_ of patients with POTS.

## Figures and Tables

**Table 1:** Basic data of the three subject groups.

Groups	Subjects			Age
	Gender	Number	Total	
POTS	M	27	60	32.31 ± 8.495
	F	33		
Healthy-Youth	M	4	13	29.3 ± 7.36
	F	9		
Healthy-Elder	M	8	10	56.5 ± 9.03
	F	2		

**Table 2:** Mean values of cardio-respiratory signals for three subject groups.

Position	Subjects	P_ETCO_2__ (mmHg)	MHR (Beat/min)	MSBP (mmHg)	MABP (mmHg)	MBR (Breath/min)	MCBFV (cm/s)
Supine (rest)	POTS	26.86 ± 3.50 ^†^	74.96 ± 11.38 ^†‡^	104.37 ± 14.12 ^†‡^	77.96 ± 9.76 ^†‡^	15.39 ± 3.85 ^†^	55.65 ± 14.01 ^‡^
	Healthy-Youth	30.84 ± 2.70 ^‡^	65.80 ± 8.56	123.75 ± 11.44	84.57 ± 8.97	17.84 ± 2.44 ^‡^	49.67 ± 15.28 ^‡^
	Healthy-Elder	28.03 ± 3.55 ^†^	64.31 ± 9.30	121.25 ± 8.22	88.45 ± 8.88	16.14 ± 2.26 ^†^	39.00 ± 11.39 ^†^
Supine (hyperventilation)	POTS	11.38 ± 2.70 ^✶^	71.77 ± 14.47	105.39 ± 15.80 ^†‡^	79.20 ± 10.94 ^†‡^	33.26 ± 4.10 ^‡^^✶^	40.98 ± 13.46 ^‡^^✶^
	Healthy-Youth	13.31 ± 3.51 ^‡^^✶^	68.43 ± 8.27	125.24 ± 13.35	83.88 ± 8.97 ^‡^	30.10 ± 4.18 ^✶^	36.13 ± 13.88 ^‡^^✶^
	Healthy-Elder	9.81 ± 4.07 ^†^	73.49 ± 15.2 ^†^^✶^	125.41 ± 13.33	91.32 ± 10.29 ^†^^✶^	29.63 ± 4.40	24.37 ± 9.64 ^†^
Tilt up	POTS	24.67 ± 4.11 ^†^^✶^	85.13 ± 18.27 ^†‡^^✶^	102.99 ± 17.88 ^†‡^	79.14 ± 12.45 ^†^	15.68 ± 5.60	46.96 ± 13.68 ^✶^
	Healthy-Youth	28.20 ± 3.30 ^✶^	71.94 ± 9.57 ^✶^	133.93 ± 16.56 ^✶^	96.95 ± 14.63	16.93 ± 2.41 ^✶^	45.51 ± 13.37 ^✶^
	Healthy-Elder	25.03 ± 4.71 ^✶^	68.20 ± 7.58 ^✶^	130.39 ± 19.17	95.67 ± 10.93 ^✶^	17.44 ± 3.15	38.68 ± 10.16 ^✶^

Note: All mean values are beat-to-beat values. MHR: mean heart rate; MSBP: mean systolic blood pressure; MABP: mean arterial blood pressure; MBR: mean breathing rate; MCBFV, mean cerebral blood flow velocity. ^†^ Significant difference compared with the Healthy-Youth (*p* < 0.05). ^‡^ Significant difference compared with the Healthy-Elder (*p* < 0.05). ^✶^ Significant difference compared with the baseline (rest) within the group (*p* < 0.05).

**Table 3:** Estimation of the logistic function parameters using curve-fitting Model I for three subject groups.

Parameters	POTS			Healthy-Youth			Healthy-Elder		
	Mean	(SD)	CV%	Mean	(SD)	CV%	Mean	(SD)	CV%
***a***, CBFV_min_ (%)	−14.02	±14.11	−100.65	−8.66	±18.13	−209.4	−13.48	±13.75	−101.97
***b***, CBFV_max_ (%)	52.53	±31.09	59.18	80.95	±28.80	35.58	88.77	±16.51	18.60
***c***, mid-P_ETCO_2__ (mmHg)	16.38	±3.48	21.24	21.83	±5.84	26.76	19.52	±7.58	38.05
***d***, P_ETCO_2__ range (mmHg)	1.47	±1.18	80.24	2.62	±1.92	73.36	1.443	±1.447	100.27
CVMR_max_ (%·mmHg^−1^)	12.65	±8.10	64.08	10.92	±6.92	63.39	31.00	±22.74	73.33

**Table 4:** Estimation of logistic function parameters using curve-fitting Model II for the three subject groups.

Parameters	POTS			Healthy-Youth			Healthy-Elder		
	Mean	(SD)	CV%	Mean	(SD)	CV%	Mean	(SD)	CV%
*a*, range of change (%)	62.42	±31.94	31.35	69.89	±39.38	36.84	92.05	±32.67	30.25
*y*_0_, CBFV_max_ (%)	49.8	±32.93	32.31	62.13	±38.66	36.16	78.52	±28.65	26.52
*x*_0_, P_ETCO_2__ level (mmHg)	19.2	±4.83	4.74	23.27	±4.58	4.28	22.37	±5.29	4.9
*b*, curvilinear (mmHg^−1^)	0.87	±0.54	0.53	0.47	±0.16	0.15	0.64	±0.59	0.55
CVMR_max_ (%·mmHg^−1^)	12.49	±9.18	9.01	7.67	±3.67	3.43	12.41	±8.47	7.84

**Table 5:** Statistical results of average fit parameters between groups for fitting Models I and II.

Regression Model	Group Comparison	Fit Parameters				
		a	b	c	d	CVMR_max_
		CBFV_min_(%)	CBFV_max_ (%)	mid-P_ETCO_2__	P_ETCO_2__ Range	
**I**	POTS vs. Healthy-Youth	*p* = 0.057	*p* = 0.011 ^✶^	*p* = 0.013 ^✶^	*p* = 0.023 ^✶^	*p* = 0.697
	POTS vs. Healthy-Elder	*p* = 0.716	*p* = 0.003 ^✶^	*p* = 0.741	*p* = 0.530	*p* = 0.038 ^✶^

^✶^ Significant distinctiveness (*p* < 0.05).

**Table 6:** Statistical results of average fit parameters between groups for fitting Models I and II.

Regression Model	Group Comparison	Fit Parameters				
		a	b	x_0_	y_0_	CVMR_max_
		Range of Change	Curvilinear	P_ETCO_2__ Level	CBFV_max_	
**II**	POTS vs. Healthy-Youth	*p* = 0.695	*p* = 0.036 ^✶^	*p* = 0.033 ^✶^	*p* = 0.466	*p* = 0.251
	POTS vs. Healthy-Elder	*p* = 0.042 ^✶^	*p* = 0.053	*p* = 0.033 ^✶^	*p* = 0.033 ^✶^	*p* = 0.676

^✶^ Significant distinctiveness (*p* < 0.05).
